# Insulin Reverses D-Glucose–Increased Nitric Oxide and Reactive Oxygen Species Generation in Human Umbilical Vein Endothelial Cells

**DOI:** 10.1371/journal.pone.0122398

**Published:** 2015-04-14

**Authors:** Marcelo González, Susana Rojas, Pía Avila, Lissette Cabrera, Roberto Villalobos, Carlos Palma, Claudio Aguayo, Eduardo Peña, Victoria Gallardo, Enrique Guzmán-Gutiérrez, Tamara Sáez, Rocío Salsoso, Carlos Sanhueza, Fabián Pardo, Andrea Leiva, Luis Sobrevia

**Affiliations:** 1 Vascular Physiology Laboratory, Department of Physiology, Faculty of Biological Sciences, Universidad de Concepción, P.O. Box 160-C, Concepción 4070386, Chile; 2 Department of Physiopathology, Faculty of Biological Sciences, Universidad de Concepción, P.O. Box 160-C, Concepción 4070386, Chile; 3 Department of Clinical Biochemistry and Immunology, Faculty of Pharmacy, Universidad de Concepción, P.O. Box 160-C, Concepción 4070386, Chile; 4 Department of Morphophysiology, Faculty of Medicine, Universidad Diego Portales, Santiago 8370076, Chile; 5 Group of Research and Innovation in Vascular Health (GRIVAS-Health), PO-Box 114-D, Chillán 3800708, Chile; 6 Faculty of Health Sciences, Universidad San Sebastián, Concepción 4080871, Chile; 7 University of Queensland Centre for Clinical Research (UQCCR), Faculty of Medicine and Biomedical Sciences, University of Queensland, Herston, QLD 4029, Queensland, Australia; 8 Department of Physiology, Faculty of Pharmacy, Universidad de Sevilla, Seville E-41012, Spain; 9 Cellular and Molecular Physiology Laboratory (CMPL), Division of Obstetrics and Gynaecology, School of Medicine, Faculty of Medicine, Pontificia Universidad Católica de Chile, P.O. Box 114-D, Santiago 8330024, Chile; University of Valencia, SPAIN

## Abstract

Vascular tone is controlled by the L-arginine/nitric oxide (NO) pathway, and NO bioavailability is strongly affected by hyperglycaemia-induced oxidative stress. Insulin leads to high expression and activity of human cationic amino acid transporter 1 (hCAT-1), NO synthesis and vasodilation; thus, a protective role of insulin on high D-glucose–alterations in endothelial function is likely. Vascular reactivity to U46619 (thromboxane A_2_ mimetic) and calcitonin gene related peptide (CGRP) was measured in KCl preconstricted human umbilical vein rings (wire myography) incubated in normal (5 mmol/L) or high (25 mmol/L) D-glucose. hCAT-1, endothelial NO synthase (eNOS), 42 and 44 kDa mitogen-activated protein kinases (p42/44^mapk^), protein kinase B/Akt (Akt) expression and activity were determined by western blotting and qRT-PCR, tetrahydrobiopterin (BH_4_) level was determined by HPLC, and L-arginine transport (0–1000 μmol/L) was measured in response to 5–25 mmol/L D-glucose (0–36 hours) in passage 2 human umbilical vein endothelial cells (HUVECs). Assays were in the absence or presence of insulin and/or apocynin (nicotinamide adenine dinucleotide phosphate-oxidase [NADPH oxidase] inhibitor), tempol or Mn(III)TMPyP (SOD mimetics). High D-glucose increased hCAT-1 expression and activity, which was biphasic (peaks: 6 and 24 hours of incubation). High D-glucose–increased maximal transport velocity was blocked by insulin and correlated with lower hCAT-1 expression and *SLC7A1* gene promoter activity. High D-glucose–increased transport parallels higher reactive oxygen species (ROS) and superoxide anion (O_2_^•–^) generation, and increased U46619-contraction and reduced CGRP-dilation of vein rings. Insulin and apocynin attenuate ROS and O_2_^•–^ generation, and restored vascular reactivity to U46619 and CGRP. Insulin, but not apocynin or tempol reversed high D-glucose–increased NO synthesis; however, tempol and Mn(III)TMPyP reversed the high D-glucose–reduced BH_4_ level. Insulin and tempol blocked the high D-glucose–increased p42/44^mapk^ phosphorylation. Vascular dysfunction caused by high D-glucose is likely attenuated by insulin through the L-arginine/NO and O_2_^•–^/NADPH oxidase pathways. These findings are of interest for better understanding vascular dysfunction in states of foetal insulin resistance and hyperglycaemia.

## Introduction

Hyperglycaemia and diabetes mellitus are pathological conditions associated with foetal endothelial dysfunction [1–4] and type 2 diabetes mellitus (T2DM) [5] or cardiovascular disease (CVD) [6, 7] in adulthood. CVD in patients with diabetes mellitus is associated with the generation of reactive oxygen species (ROS) [8] caused by chronic hyperglycaemia [3] and insulin resistance [9]. Nicotinamide adenine dinucleotide phosphate-oxidase (NADPH oxidase) activity and endothelial nitric oxide (NO) synthase (eNOS) uncoupling leads to vascular ROS generation [10], of which superoxide anion (O_2_^•–^) reduces NO bioavailability, generating peroxynitrite (ONOO^–^) and resulting in altered vascular endothelial function [11]. NO is synthesised by eNOS from the cationic amino acid L-arginine, which is taken up from the extracellular space by the human cationic amino acid transporter 1 (hCAT-1), a member of the cationic amino acid transporter (CATs) family [11], in human umbilical vein endothelial cells (HUVECs) [12]. Thus, NO bioavailability depends on eNOS activity and hCAT-1 expression and activity [13], as well as ROS levels [11], in this cell type.

Elevated extracellular D-glucose increases hCAT-1-mediated L-arginine transport and NO synthesis (the ‘L-arginine/NO pathway’) [14, 15] as well as intracellular ROS generation [16, 17], leading to endothelial dysfunction [2]. Insulin causes human umbilical vein endothelium-dependent dilation and increases hCAT-1 expression caused by elevated *SLC7A1* (encoding hCAT-1) transcriptional activity [12, 14, 18] and NO synthesis [12]. Additionally, insulin reverses the gestational diabetes mellitus (GDM) or high D-glucose-associated stimulation of the L-arginine/NO pathway in HUVECs [11]. We hypothesise that insulin has a beneficial antioxidant capacity that reverses the high D-glucose-associated increase in ROS generation. We studied the effect of high extracellular D-glucose on insulin modulation of the L-arginine/NO and NADPH oxidase/O_2_^•–^ pathways in fetoplacental vascular reactivity. The results suggest that D-glucose-increased oxidative stress and vascular dysfunction are attenuated by insulin. Thus, insulin likely acts as an antioxidant under conditions of hyperglycaemia, leading to protection of the fetoplacental endothelium in diseases associated with endothelial dysfunction such as GDM.

## Methods

### Ethics statement

This investigation conforms to the principles outlined in the Declaration of Helsinki and has received approval from the Ethics Committee of the Faculty of Medicine of the Pontificia Universidad Católica de Chile, the Faculty of Biological Sciences of Universidad de Concepción, and the Comisión Nacional de Investigación en Ciencia y Tecnología (CONICYT grant numbers 1110799 and 11100192, Chile). Patient-informed written consent forms were obtained 12–24 hours before delivery.

### Human placenta and cell culture

Placentas with their umbilical cords were collected after delivery from 33 full-term normal pregnancies from the Hospital Clínico Universidad Católica in Santiago (Chile) and from the Hospital Regional Guillermo Grant Benavente in Concepción (Chile). All of the pregnancies were single births. The pregnant women did not smoke or consume drugs or alcohol and had no intrauterine infection or any medical or obstetrical complications. The women were normotensive and have normal weight and exhibited a normal response to the oral glucose tolerance test. They were under a normal food regimen during the entire pregnancy period. In addition, the new-borns (46% female, 54% male) were born at term by vaginal delivery and were of normal birth weight and height.

The placentas were transported in a sterile container (4°C) to the laboratory. Sections of umbilical cords (100–120 mm length) were collected into sterile 200 mL phosphate-buffered saline (PBS) solution ((mmol/L): 130 NaCl, 2.7 KCl, 0.8 Na_2_HPO_4_, 1.4 KH_2_PO_4_ (pH 7.4, 4°C)) and used for the isolation of HUVECs between 6–12 hours after delivery [12].

The HUVECs were isolated by collagenase digestion (0.25 mg/mL Collagenase Type II from *Clostridium histolyticum*; Invitrogen, Carlsbad, CA, USA) as previously described [12]. The cells were cultured (37°C, 5% CO_2_) to passage 3 in medium 199 (M199) (Gibco Life Technologies, Carlsbad, CA, USA) containing 5 mmol/L D-glucose, 10% new-born calf serum (NBCS), 10% foetal calf serum (FCS), 3.2 mmol/L L-glutamine, and 100 U/mL penicillin-streptomycin (primary culture medium, PCM). The experiments were performed on the cells incubated (0–24 hours) in PCM containing increasing concentrations of D-glucose (5, 10, 15, 20 or 25 mmol/L) in the absence or presence (8 hours) of insulin (0.001, 0.01, 0.1, 1 or 10 nmol/L). In the experiments in which the time of exposure to D-glucose was longer than 8 hours, insulin was added for the final 8 hours of incubation. In some experiments, the cells were incubated with 5 mmol/L D-glucose + 20 mmol/L D-mannitol or 20 mmol/L L-glucose. The cell viability estimated by Trypan blue exclusion was >97%. Sixteen hours prior to the experiments, the incubation medium was changed to serum-free M199 [12].

### L-Arginine transport

The overall L-arginine transport (3 μCi/mL L-[^3^H]arginine (NEN, Dreieich, FRG), 0–1000 μmol/L L-arginine, 1 minute, 37°C) was measured as described [12, 14, 18]. Briefly, the transport assays were performed in Krebs solution ((mmol/L): NaCl 131, KCl 5.6, NaHCO_3_ 25, NaH_2_PO_4_ 1, HEPES 20, CaCl_2_ 2.5, MgCl_2_ 1 (pH 7.4, 37°C)) in cells preincubated (12 hours) in PCM containing 2% sera and different concentrations of D-glucose, 5 mmol/L D-glucose + 20 mmol/L D-mannitol or 5 mmol/L D-glucose + 20 mmol/L L-glucose. The cell monolayers were rinsed with ice-cold Krebs solution to terminate the tracer uptake. The radioactivity in the formic acid cell digests was determined by liquid scintillation counting, and the uptake was corrected for D-[^3^H]mannitol (NEN) disintegrations per minute (d.p.m.) in the extracellular space [12].

The overall transport at initial rates (i.e., the linear uptake up to 1 minute) was adjusted to the Michaelis-Menten hyperbola with a nonsaturable, lineal component as described [12, 18]. The maximal velocity (*V*_max_) and apparent Michaelis-Menten constant (*K*_m_) of saturable transport were calculated as described [12, 14, 18].

### Western blotting

Total protein was obtained from the confluent cells, washed twice with ice-cold PBS and harvested in 100 μL of lysis buffer (63.7 mmol/L Tris/HCl (pH 6.8), 10% glycerol, 2% sodium dodecyl sulphate, 1 mmol/L sodium orthovanadate, 50 mg/mL leupeptin, 5% 2-mercaptoethanol) as described [12]. The cells were sonicated (six cycles, 5 seconds, 100 W, 4°C), and total protein was separated by centrifugation (12,000 rpm, 15 minutes, 4°C). The proteins (50 μg) were separated by polyacrylamide gel (8–10%) electrophoresis and transferred to Immobilon-P polyvinylidene difluoride membranes (BioRad Laboratories, Hertfordshire, UK). The proteins were then probed with primary monoclonal mouse *anti*-hCAT-1 (1:1500, 12 hours, 4°C) (Sigma-Aldrich, St. Louis, MO, USA), primary polyclonal goat *anti*-total and phosphorylated mitogen-activated 42 and 44 kDa protein kinases (p42/44^mapk^, 1:1000) or protein kinase B/Akt (Akt, 1:1000) (Cell Signalling, Danvers, MA, USA) or monoclonal mouse *anti*-ß-actin (1:2000, 1 hour, room temperature) (Santa Cruz Biotechnology, Santa Cruz, CA, USA) antibodies. The membranes were rinsed in Tris-buffered saline with Tween (TBS-T) and incubated (1 hour) in TBS-T/0.2% bovine serum albumin (BSA) containing secondary horseradish peroxidase-conjugated goat *anti*-mouse (AbCam, Cambridge, MA, USA) or *anti*-goat (Santa Cruz Biotechnology) antibodies. Proteins were detected by enhanced chemiluminescence (film exposure time was 2 min) in a ChemiDoc-It 510 Imagen System (UVP, LCC Upland, CA, USA) and quantified by densitometry as described [12].

### Immunofluorescence and confocal laser scanning microscopy

The HUVECs were grown on microscope cover slips (10^6^ cells/slide) (Marienfeld GmbH & Co. KG, LaudaKönigshofen, Baden-Württemberg, Germany) in PCM to 90% confluence. The cells were cultured for 24 hours in PCM containing 25 mmol/L D-glucose in the absence or presence of 1 nmol/L insulin (see above). The cells were then fixed in 4% paraformaldehyde (15 minutes), rinsed (x3) with Hanks solution ((mmol/L): CaCl_2_ 1.26, KCl_2_ 5.37, KH_2_PO_4_ 0.44, MgSO_4_ 8.11, NaCl 136.89, Na_2_HPO_4_ 0.33, NaHCO_3_ 4.16 (37°C, pH 7.4)), permeabilized with 0.1% Triton X-100 (20 minutes), and blocked (1 hour) with 1% BSA. hCAT-1 was immunolocalized by incubating the cells with primary polyclonal rabbit *anti-*hCAT-1 (1:1500, overnight at 4°C) in PBS containing 5% BSA. The fixed cells were then washed (x3) with Hanks solution followed by incubation (1 hour) with the secondary antibody, fluorescein isothiocyanate (FITC) goat *anti*-mouse IgG (H+L) (λ_exc_/λ_em_:492/520 nm) (1:2000) (Thermo Fisher Scientific, Inc., Waltham, MA, USA) in PBS containing 5% BSA. The nuclei were counterstained with Vectashield mounting medium and stained with 4’,6-diamidino-2-phenylindole (DAPI) (Vector Laboratories, Burlingame, CA, USA). The samples were analysed under an Olympus IX81 microscope with a disk scanning unit (DSU) spinning disk confocal system (Olympus, Tokyo, Japan). The images were obtained with a Hamamatsu ORCA-R2 camera (Hamamatsu Photonics, Hamamatsu, Japan) controlled by the Olympus XcellenceR software using a Plan Apo N 60x 1.42 NA objective. Each sample was examined through successive 0.2 μm optical slices along the z axis, and the obtained images were post-processed by applying a Wiener filter deconvolution provided by the Olympus software.

### Total RNA isolation and reverse transcription

Total RNA was isolated using the Chomczynski method as previously described [12]. The RNA quality and integrity were ensured by gel visualisation and spectrophotometric analysis (OD_260/280_), quantified at 260 nm and precipitated to obtain 4 μg/μL RNA. Aliquots (1 μg) of the total RNA were reverse transcribed into cDNA as described [14, 18, 19].

### Quantitative RT-PCR

The experiments were performed using a LightCycler rapid thermal cycler (Roche Diagnostics, Lewes, UK) in a reaction mixture containing 0.5 μmol/L primers, dNTPs, *Taq* DNA polymerase and reaction buffer provided in QuantiTectSYBR Green PCR Master Mix (QIAGEN, Crawley, UK) as described [8, 18, 19]. Hot Start *Taq* DNA polymerase was activated (15 minutes, 95°C), and the assays included 95°C denaturation (15 seconds), annealing (20 seconds) at 54°C (hCAT-1 and 28S), and extension (10 seconds) at 72°C (hCAT-1 and 28S). The product melting temperatures were 79.1°C (hCAT-1) and 86.7°C (28S). The following oligonucleotide primers were used: hCAT-1 (sense) 5’-GAGTTAGATCCAGCAGACCA-3’, hCAT-1 (*anti*-sense) 5’-TGTTCACAATTAGCCCAGAG-3’, 28S (sense) 5’-TTGAAAATCCGGGGGAGAG-3’, 28S (*anti*-sense) 5’-ACATTGTTCCAACATGCCAG-3’. The number of copies of *28S* rRNA was not significantly altered (*P >* 0.05, *n* = 4) in any experimental conditions used in this study.

### Actinomycin D effect on hCAT-1 mRNA

Total RNA and protein were isolated from the HUVECs cultured in PCM containing 5 or 25 mmol/L D-glucose (24 hours) in the absence or presence (8 hours) of 1.5 μmol/L actinomycin D (transcription inhibitor) [20] and/or 1 nmol/L insulin. The *hCAT-1* mRNA and 28S rRNA were quantified by real-time RT-PCR.

### hCAT-1 promoter cloning

The upstream sequences -1606 and -650 bp from the transcription start codon of the *SLC7A1* gene (GenBank: AL596114) were PCR-amplified and cloned into the pGL3-basic reporter system to generate the pGL3-hCAT1^-1606^ and pGL3-hCAT1^-650^ reporter constructs as described [12, 18, 19].

### Transient transfection

Sub-confluent (60–80%) HUVECs primary cultures were resuspended in serum-free M199. Aliquots of the cell suspension (0.5 mL, 3.2 x10^6^ cells/mL) were mixed with 10 μg of the pGL3-hCAT1^-1606^ or pGL3-hCAT1^-650^ constructs, pGL3-Basic (empty pGL3 vector), pGL3-Control (Simian Virus 40 promoter (SV40) pGL3 vector), and the internal transfection control vector, pRL-TK expressing *Renilla* luciferase (Promega) [19, 20]. After electroporation (300 Volts, 700 μF, 5–10 milliseconds) (Gene Pulser II System, BioRad, CA, USA), the cells were cultured (49 hours) in M199 containing 2% FCS. The transfection efficiency was estimated by transfection of the pEGFP-N3 vector (Clontech, Mountain View, CA, USA), and the fluorescent cells were counted under an inverted fluorescent microscope (Leica DMIL; Wetzlar, Germany) [19, 20].

### Luciferase assay

The electroporated cells were lysed in 200 μL of passive lysis buffer (Promega), and firefly and *Renilla* luciferase activity were measured using the Dual-Luciferase Reporter Assay System (Promega) in a Sirius luminometer (Berthold Detection System; Oak Ridge, TN, USA) [19, 20].

### Intracellular ROS and NO

Intracellular ROS and NO levels were determined using the fluorescent dyes 5-(and-6)-chloromethyl-2',7'-dichlorodihydrofluorescein diacetate (CM-H_2_DCFDA) and 4-amino-5-methylamino-2',7'-difluorofluorescein (DAF-FM) (Molecular Probes, Leiden, The Netherlands), respectively. Confluent cells in a 100 mm^2^ plate were incubated with high D-glucose (25 mmol/L, 24 hours) and/or insulin (1 nmol/L, 8 hours) and/or 1-(4-hydroxy-3-methoxyphenyl)-ethanone (apocynin, 100 μmol/L, 24 hours, NADPH oxidase inhibitor) and exposed (45 minutes, 37°C) to 10 μmol/L of CM-H_2_DCFDA or DAF-FM in PBS (37°C, pH 7.4). The fluorescence (λ_exc_/λ_em_: 495/510 nm) was determined in a Sinergy 2 (Biotek, Winooski, VT, USA) microplate reader [21].

### Tetrahydrobiopterin (BH_4_) determination by HPLC

The level of BH_4_ was determined by an acid-base oxidation method followed by fluorometric detection by high performance liquid chromatography (HPLC). The total biopterins level (BH_4_ + dihydrobiopterin (BH_2_) + biopterins) were determined by acid oxidation and BH_2_ + biopterins levels by basic oxidation, following a modification of the method described by Fukushima & Nixon [22]. Confluent HUVECs in 100 mm diameter culture plates in 5 or 25 mmol/L glucose in the absence or presence (24 hours) of 1 nmol/L insulin, 100 μmol/L apocynin, 100 μmol/L Mn(III)tetrakis(1-methyl-4-pyridyl)porphyrin pentachloride (Mn(III)TMPyP), or 1 mmol/L tempol were collected in cold PBS (4°C). Cells were then centrifuged (1,000 *g*, 2 minutes, 4°C) and lysed in 200 μL of biopterins lysis buffer ((mmol/L): 50 Tris-HCl (pH 7.4), 2 dithiothreitol (DTT), 1 ethylenediamine tetraacetic acid (EDTA)). Cells were then sonicated (tree cycles, 10 seconds, 100W, 4°C) and centrifuged (13,500 *g*, 20 minutes, 4°C). Proteins were quantified in 20 μL of the supernatants by a modified Lowry method (Bio-Rad DC protein assay, BioRad Laboratories) as described [12]. Proteins contained in 180 μL of supernatant were precipitated by mixing with 20 μL of 1.5 M HClO_4_:2 M H_3_PO_4_ = 1:1 (v/v) by strong vortex (5 seconds), allowing 30 minutes (4°C) incubation and further centrifugation (13,500 *g*, 5 minutes, 4°C). Aliquots of 90 μL of the protein-free supernatant were used for total biopterins determination by acid oxidation with 10 μL of iodine/KI acid solution (1% iodine in a 2% KI solution prepared in 1 M HCl). For BH_2_ + biopterins determination an alkali oxidation was performed by mixing 10 μL of 1 M NaOH with 80 μL of protein-free supernatant, followed by addition of 10 μL of iodine/KI alkali solution (1% iodine in a 2% KI solution prepared in 1 M NaOH). Samples for total biopterins and BH_2_ + biopterins determination were incubated at 23°C for 1 hour in darkness. After this incubation period iodine in the samples was reduced by adding 5 μL of fresh ascorbic acid (20 mg/mL). Samples for BH_2_ + biopterins determination were acidified by mixing with 20 μL of 1 M H_3_PO_4_. All samples (i.e., acid and alkali oxidation) were centrifuged (13,500 *g*, 5 minutes, 23°C), passed through a Millex syringe-driven filter unit (Millipore Corporation, Billerica, MA, USA) and loaded in an analytical column (C_18_: 4.6 mm x 150 mm) (HiQsil, KYA Tech, Japan) coupled to a HPLC system (PU2089S, Jasco, Japan). The analytical column was washed by passing through ultrapure water (Milli-Q, Millipore Corporation) (pH 7.3, 23°C, 20 minutes) and further washed with methanol (>99%, 20 minutes). The column was then equilibrated with the mobile phase (5% methanol, 95% water) for 40 minutes with a flow rate of 1 mL/minute. Fluorescence was monitored at excitation and emission wavelengths of 350 and 450 nm, respectively, by using the fluorescent detector (FP 2020 Plus, Jasco, Japan) of the HPLC system. Biopterins were quantified against the standard curve 0–100 nmol/L L-biopterin (Sigma-Aldrich). The chromatograms were obtained and analysed by using the software ChromPass 1.7 (ChromPass Chromatography Data System, Jasco, Japan). BH_4_ was determined as the difference between the areas under the curve in chromatograms for total biopterins and BH_2_ + biopterin. Values for BH_4_ level are given in pmol/μg protein.

### O_2_^•–^ level

Intracellular O_2_^•–^ was quantified using lucigenin (Sigma-Aldrich) [23]. Confluent cells in 100 mm^2^ plates were incubated with high D-glucose (25 mmol/L, 24 hours) in the absence or presence of insulin (1 nmol/L), apocynin (100 μmol/L, 24 hours) and/or 4-hydroxy 2,2,6,6,-tetramethyl piperidine 1-oxyl (tempol; 1 mmol/L, 24 hours, superoxide dismutase (SOD) mimetic), harvested, and centrifuged (1500 rpm, 5 minutes, 4°C), and the cellular pellet was resuspended and washed with PBS (pH 7.4, 4°C). A volume of 50 μL of resuspended cells was stabilised (5 minutes, 25°C) and incubated with lucigenin (10 μmol/L, 30 minutes). The photon emission was determined every 15 seconds for 10 minutes in a Sirius luminometer (Berthold Detection System; Oak Ridge, TN, USA) [23].

### Umbilical vein reactivity

Ring segments (2–4 mm length) from human umbilical veins were mounted in a myograph (610M Multiwire Myograph System, Danish Myo Technology A/S, Denmark) for isometric force measurements with the optimal diameter adjusted from the maximal active response to 90 mmol/L KCl, as described [12]. The vessels were incubated (24 hours, 37°C) with 5 or 25 mmol/L D-glucose, 5 mmol/L D-glucose + 20 mmol/L D-mannitol, 100 μmol/L apocynin, 1 nmol/L insulin and/or 1 mmol/L tempol. After this incubation period, the response to increasing concentrations of U46619 (0.1–1000 nmol/L, 5 minutes) (thromboxane A_2_ analogue) [12, 24] or calcitonin gene related peptide (CGRP, 0.001–100 nmol/L, 5 minutes) [12] (Sigma-Aldrich) was assayed.

### Statistical analysis

The values are the mean ± S.E.M. for different cell cultures (with 3–4 replicates) from an equal number of placentas (*n* = 33). The data reported in this study describe a normal standard distribution. The comparisons between two or more groups were performed using Student’s unpaired *t*-test and analysis of variance (ANOVA), respectively. If the ANOVA demonstrated a significant interaction between the variables, *post hoc* analyses were performed by the multiple-comparison Bonferroni correction test. The GraphPad Instat 3.0b and GraphPad Prism 6.0f (GraphPad Software, Inc., San Diego, CA, USA) statistical software packages were used for the data analysis. *P*<0.05 was considered significant.

## Results

### High D-glucose increases L-arginine uptake and hCAT-1 expression

**Fig 1:**
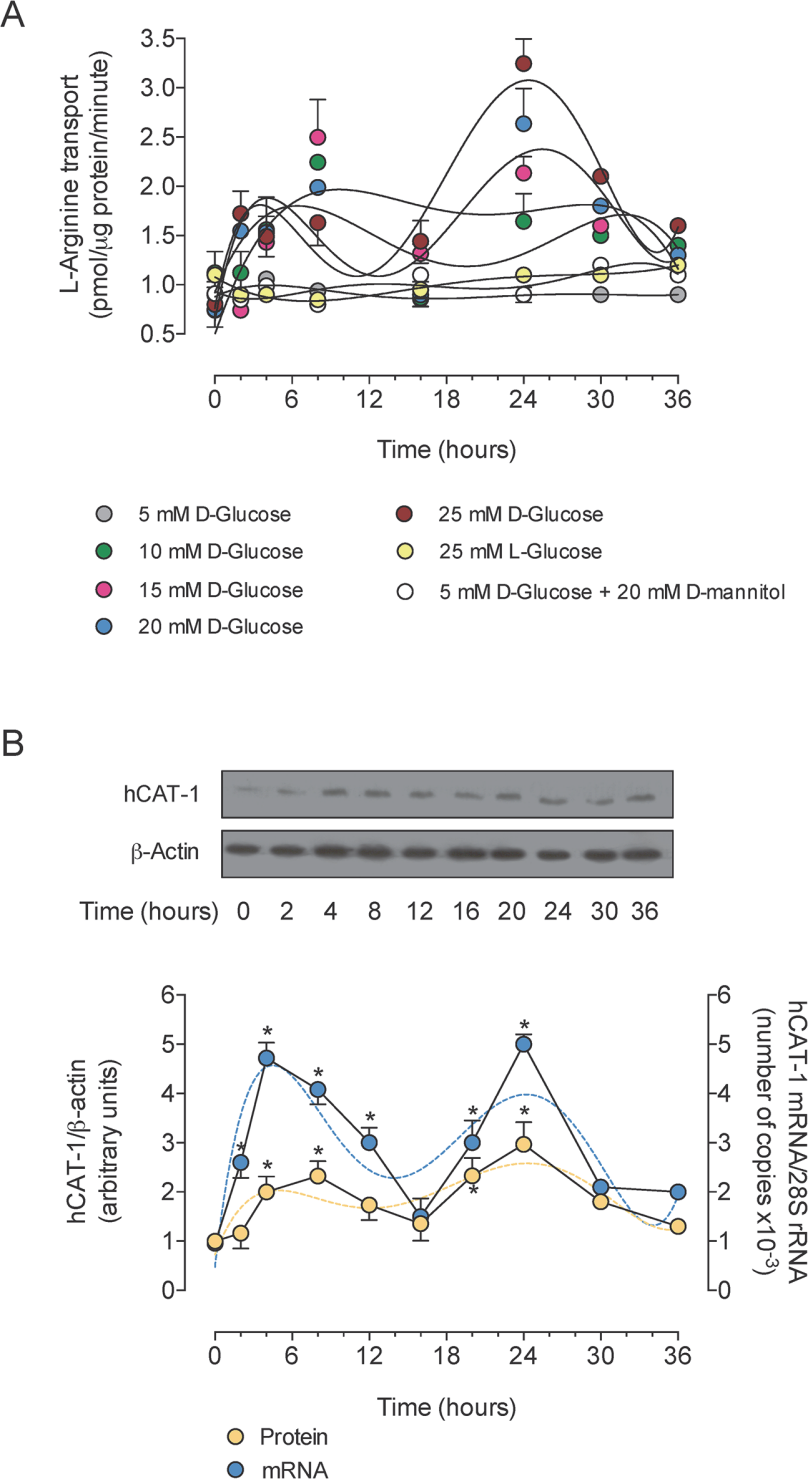
Temporal increase of hCAT-1 expression and activity by high D-glucose. A, Uptake of 100 μmol/L L-arginine (2 μCi/mL L-[^3^H]arginine, 1 minute, 37°C) in HUVECs preincubated in Krebs solution containing different concentrations of D-glucose, or 5 mmol/L D-glucose + D-mannitol or L-glucose. B, hCAT-1 protein abundance and mRNA expression in 25 mmol/L D-glucose. Western blot is representative of other 10 experiments for hCAT-1 and β-actin (internal reference) proteins. In A lines are the mean values adjusted to a fifth-order polynomial model. In B the segmented lines represent the mean values for protein abundance (light blue) or mRNA expression (yellow) as in A. *P<0.04 versus corresponding values at 0 hours. Values are mean ± S.E.M. (*n* = 5–10).

High D-glucose increased L-arginine transport in a concentration- and time-dependent manner (Fig 1A). The D-glucose effect was biphasic, with an initial increase from 2 hours of incubation reaching a maximal first increase at 8 hours of incubation. The L-arginine transport was unaltered at 16 hours, but it increased at 24 hours of incubation. The kinetics for a primary peak half-stimulatory effect (^*pp*^ *SE*_50_) were similar for 10 and 15 mmol/L as well as for 20 and 25 mmol/L D-glucose (Table 1). The ^*pp*^ *SE*_50_ values for 10 and 15 mmol/L were higher than the values for 20 and 25 mmol/L D-glucose. However, the kinetics for the secondary peak half-stimulatory effect (^*sp*^ *SE*_50_) was similar at all D-glucose concentrations. The half-maximal stimulatory concentration (*SC*_50_) of D-glucose at 24 hours of incubation on L-arginine transport was 14.1 ± 2.1 mmol/L. Transport was unaltered in cells incubated with 5 mmol/L D-glucose + 20 mmol/L D-mannitol or 5 mmol/L D-glucose + 20 mmol/L L-glucose (Fig 1A). Because a significant increase in transport by 25 mmol/L D-glucose (hereafter referred as ‘high D-glucose’) was observed starting at approximately 2 hours of incubation and maintained for at least 24 hours, we assayed the insulin effect in the cells incubated with this concentration of D-glucose for 24 hours. Additionally, *hCAT-1* mRNA expression and protein abundance increased in a biphasic manner upon high D-glucose treatment (Fig 1B). The ^*pp*^ *SE*_50_ and ^*sp*^ *SE*_50_ values of *hCAT-1* mRNA expression were lower than the corresponding values for hCAT-1 protein abundance (Table 1).

**Table 1:** Effect of D-glucose on L-arginine transport in HUVECs.

*Change in 100 μmol/L L-arginine uptake*			
	^*pp*^ *SE*_50_ (hours)	^*sp*^ *SE*_50_ (hours)	^*pp*^ *SE*_50_/^*sp*^ *SE*_50_
D-Glucose 5	–	–	–
D-Glucose 10	3.9 ± 0.3	23 ± 0.5	5.9 ± 0.3
D-Glucose 15	4.1 ± 0.2	23 ± 0.4	5.6 ± 0.2
D-Glucose 20	1.9 ± 0.2*	22 ± 0.4	11.6 ± 0.7*
D-Glucose 25	2.9 ± 0.1*	20.8 ± 0.5	7.2 ± 0.1*
D-Glucose 5 + L-glucose 20	–	–	–
D-Glucose 5 + D-mannitol 20	–	–	–
*Change in hCAT-1 mRNA expression*			
D-Glucose 25	1.7 ± 0.1	22.2 ± 0.2	13.1 ± 0.5
*Change in hCAT-1 protein abundance*			
D-Glucose 25	3.0 ± 0.2†	22.5 ± 0.3†	7.5 ± 0.3
*L-Arginine transport kinetics*			
	*V*_max_ (pmol/μg protein/minute)	*K*_m_ (μmol/L)	*V*_max_/*K*_m_ (pmol/μg protein/minute/(μmol/L))
D-Glucose 5	2.6 ± 0.3	123 ± 43	0.021 ± 0.005
D-Glucose 25	10.3 ± 0.7*	196 ± 36	0.053 ± 0.011*
D-Glucose 25 + insulin 0.01	9.8 ± 1.2*	150 ± 59	0.065 ± 0.017*
D-Glucose 25 + insulin 0.1	6.7 ± 0.5*	122 ± 28	0.055 ± 0.009*
D-Glucose 25 + insulin 1	1.7 ± 0.2†	117 ± 58	0.015 ± 0.005†
D-Glucose 25 + insulin 10	3.0 ± 0.2†	127 ± 28	0.024 ± 0.003†
D-Glucose 5 + L-glucose 20 + insulin 1	8.1 ± 0.6†	121 ± 36	0.067 ± 0.012†
D-Glucose 5 + D-mannitol 20 + insulin 1	8.3 ± 0.8†	136 ± 41	0.061 ± 0.012†

L-Arginine uptake (100 μmol/L) in HUVECs exposed (0–24 hours) to Krebs solution with increasing concentrations of D-glucose, or 5 mmol/L D-glucose + 20 mmol/L L-glucose or 20 mmol/L D-mannitol (see Methods). Kinetics of saturable L-arginine transport (0–1000 μmol/L L-arginine, 2 μCi/mL L-[^3^H]arginine, 1 minute, 37°C) in cells exposed (24 hours) to Krebs solution containing 5 or 25 mmol/L D-glucose with insulin (0.01–10 nmol/L) (8 hours), or 5 mmol/L D-glucose + 20 mmol/L L-glucose or 20 mmol/L D-mannitol (see Methods). *V*_max_/*K*_m_: maximal transport capacity, ^pp^ *SE*_50_: primary peak half-stimulatory D-glucose effect, ^sp^ *SE*_50_: secondary peak half-stimulatory D-glucose effect, hCAT-1: human cationic amino acid transporter 1.—denotes not significant change.

*P<0.05 versus 5 mmol/L D-glucose

^†^ *P*<0.05 versus 25 mmol/L D-glucose. Values are means ± S.E.M. (n = 10–12).

### Insulin blocks D-glucose–increased hCAT-1 activity and expression

**Fig 2:**
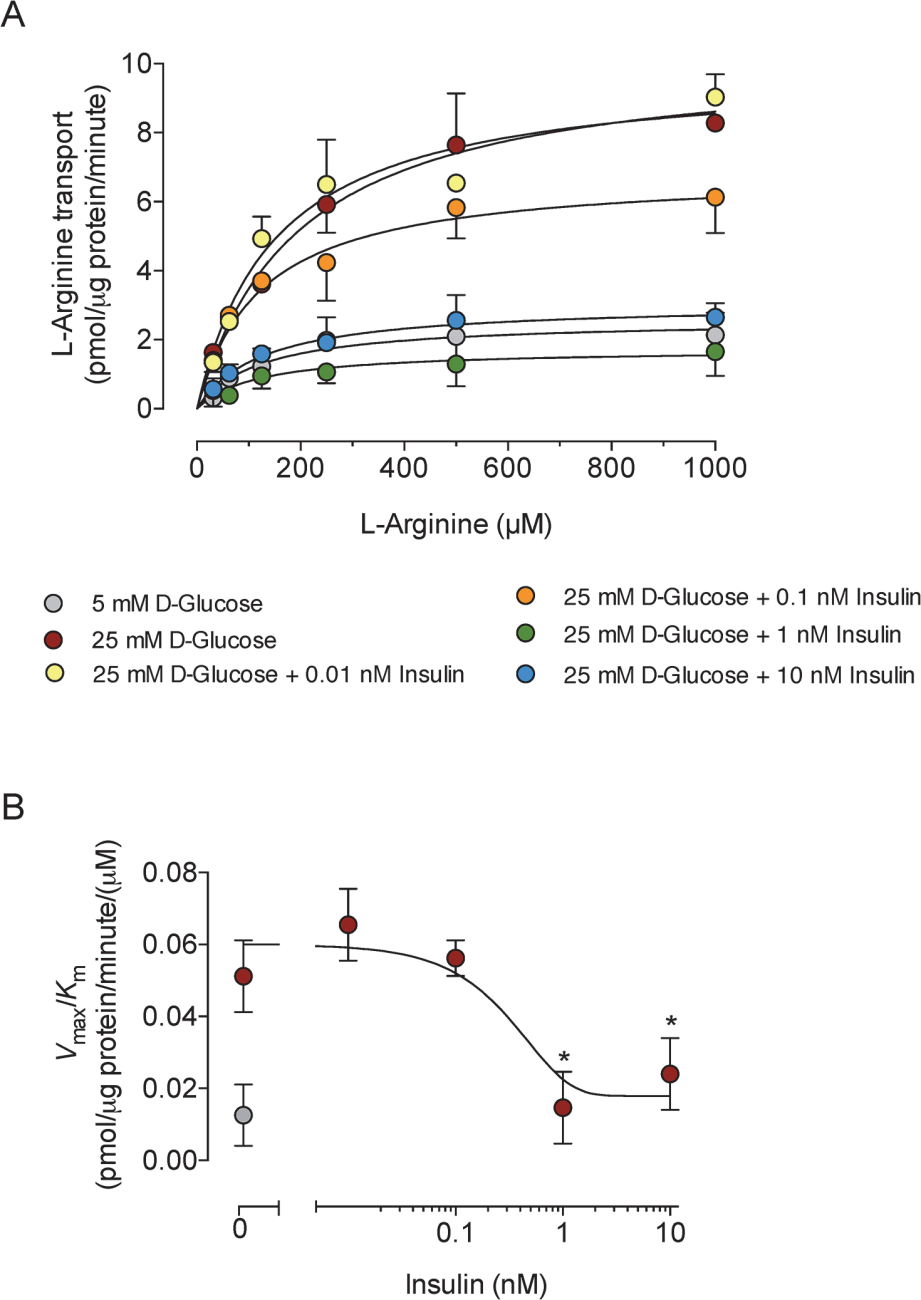
Insulin modulation of kinetic parameters for saturable L-arginine transport in response to high D-glucose. A, Saturable L-arginine transport (0–1000 μmol/L L-arginine, 2 μCi/mL L-[^3^H]arginine, 1 minute, 37°C) in HUVECs preincubated (24 hours) in Krebs solution containing 5 mmol/L D-glucose (control) or 25 mmol/L D-glucose + insulin. B, Maximal transport capacity (*V*_max_/*K*_m_) values for saturable L-arginine transport from data in A. *P<0.04 versus values in the absence of insulin. Values are mean ± S.E.M. (*n* = 12–15).

We previously reported that insulin increases the *V*_max_ of L-arginine transport, without altering the apparent *K*_m_, in a concentration-dependent manner in HUVECs cultured in 5 mmol/L D-glucose [12]. The high D-glucose effect was reflected in a higher *V*_max_/*K*_m_, with a maximal transport increase at 1 nmol/L insulin [12]. Insulin blocked the increase in L-arginine transport caused by high D-glucose in a concentration-dependent manner by decreasing the *V*_max_ (Fig 2A) and *V*_max_/*K*_m_ (Table 1) values of transport, with a half-maximal inhibitory concentration (*IC*_50_) for an insulin effect of 0.11 ± 0.007 nmol/L (Fig 2B).

**Fig 3:**
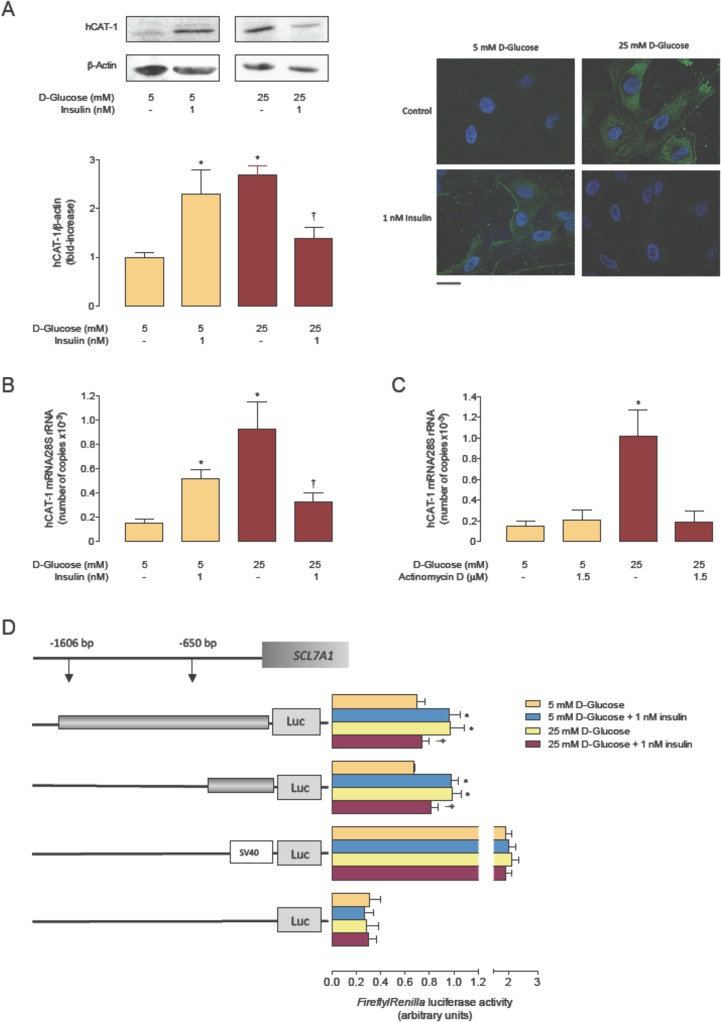
Insulin blocks high D-glucose-increase in hCAT-1 expression. A, Western blot for hCAT-1 protein abundance in whole HUVECs extracts incubated (24 hours) in 5 or 25 mmol/L D-glucose in the absence (–) or presence of insulin. *Lower panel*: hCAT-1/β-actin ratio densitometries normalized to 1 in 5 mmol/L D-glucose in the absence of insulin. β -Actin is internal reference. B, Immunocytochemistry of hCAT-1 (green fluorescence) in cells as in A. Control are cells in the absence of insulin. Bar indicates 5 μm at 60x microscopy magnification. C, Number of copies of *hCAT-1* mRNA and *28S* rRNA (internal reference) in cells as in A. D, Number of copies of hCAT-1 mRNA and *28S* rRNA in cells incubated (24 hours) with 5 or 25 mmol/L D-glucose in the absence (–) or presence of actinomycin D. E, Luciferase reporter construct containing two truncations of *SLC7A1* promoter (-1606 bp (pGL3-hCAT-1^-1606^) and -650 bp (pGL3-hCAT-1^-650^) from ATG) were transfected in HUVECs primary cultures as in A. In A, C and E, *P<0.04 or †*P*<0.05 versus 5 mmol/L D-glucose in the absence of insulin. In C, *P<0.03 versus all other values. Values are mean ± S.E.M. (*n* = 5).

Insulin restored the increase in hCAT-1 protein abundance (Fig 3A), *hCAT-1* mRNA expression (Fig 3B) and fluorescence (Fig 3C). In addition, high D-glucose and insulin increased these parameters in cells in 5 mmol/L D-glucose. Actinomycin D blocked the effect of high D-glucose on *hCAT-1* mRNA expression (Fig 3D). In addition, incubation of cells with high D-glucose increased *SLC7A1* promoter transcriptional activity in cells transfected with either pGL3-hCAT1^-650^ (1.39 ± 0.08-fold) or pGL3-hCAT1^-1606^ (1.46 ± 0.05-fold) constructs compared with non-transfected cells in 5 mmol/L D-glucose (Fig 3E). Insulin increased the transcriptional activity only in cells transfected with pGL3-hCAT1^-1606^ (1.37 ± 0.06-fold). However, insulin reverted the effect of high D-glucose on *SLC7A1* transcriptional activity for cells containing each construct.

### Insulin blocks D-glucose–increased ROS and NO synthesis

**Fig 4:**
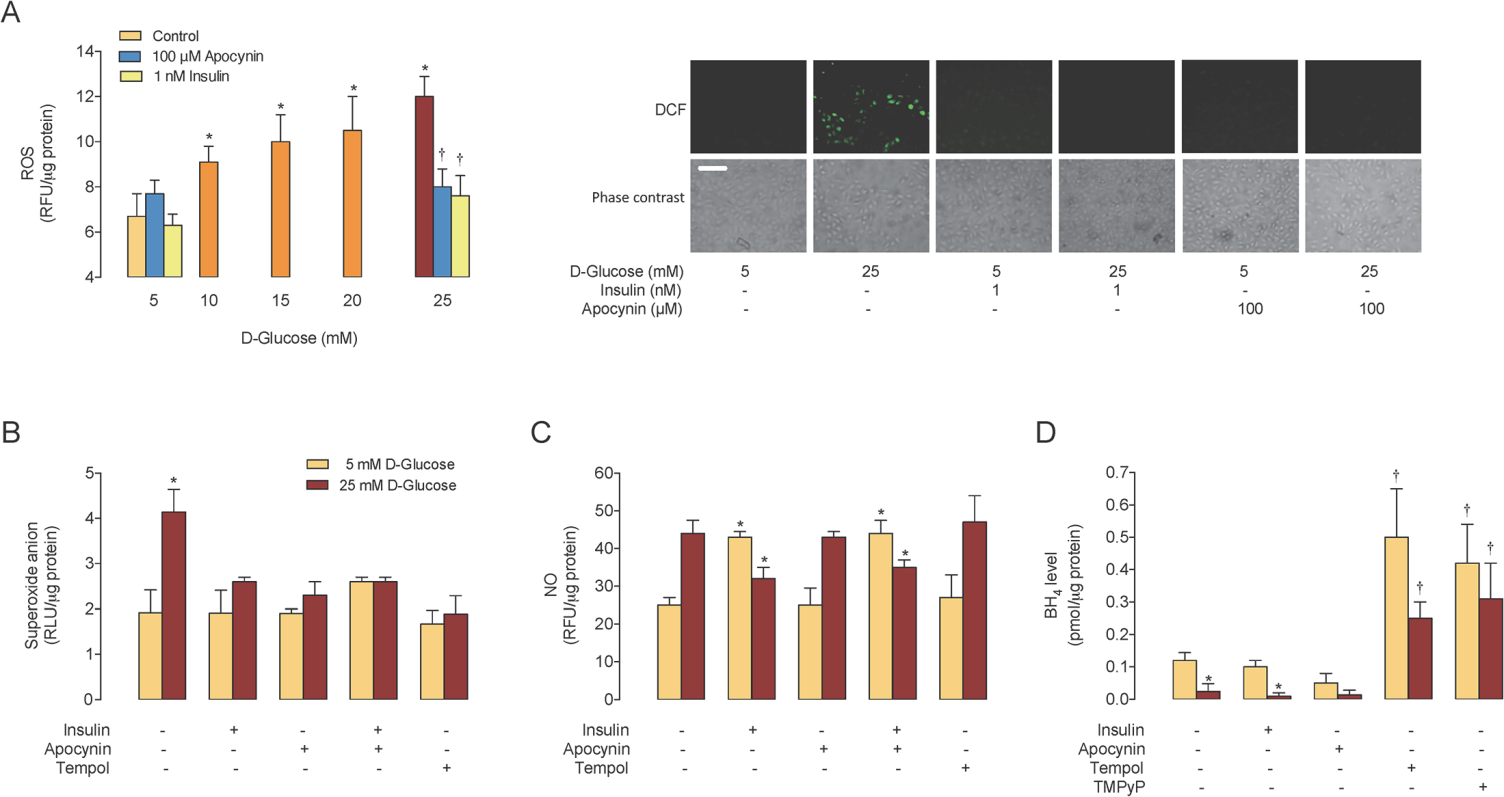
High D-glucose–modulation of ROS, O_2_^•–^ and BH4 generation. A, Reactive oxygen species (ROS) in HUVECs pre-incubated (24 hours) with D-glucose at different concentrations. Relative fluorescence units (RFU) was measured in cells preloaded (30 minutes) with 5-(and-6)-chloromethyl-2',7'-dichlorodihydrofluorescein diacetate (DCF) in the absence or presence of apocynin or insulin. Control is fluorescence in cells in 5 mmol/L D-glucose in the absence of insulin or apocynin. *Left panel*. ROS level (as DCF fluorescence) in the absence (–) or presence (+) of apocynin or insulin in cells incubated with 5 or 25 mmol/L D-glucose. Bar indicates 200 μm at 10x microscopy magnification. B, The superoxide anion (O_2_^•–^) level in cells pre-incubated (24 hours) with 5 or 25 mmol/L D-glucose in the absence (–) or presence (+) of 1 nmol/L insulin, 100 μmol/L apocynin or 1 mmol/L tempol, measured as relative luminescence units (RLU) in cells preloaded (30 minutes) with 10 μmol/L lucigenin. C, Nitric oxide (NO) level in cells as in B, measured as RFU in cells preloaded (30 minutes) with 4-amino-5-methylamino-2',7'-difluorofluorescein. D, BH_4_ level measured by HPLC in cells as in B, and in the absence or presence of 100 μmol/L Mn(III)TMPyP (TMPyP). In A, *P<0.03 versus Control. †*P*<0.04 versus corresponding values in 25 mmol/L D-glucose. In B, *P<0.03 versus all other values. In C, *P<0.03 versus corresponding values in the absence of insulin, apocynin or tempol. In D, *P<0.05 versus corresponding values in 5 mmol/L D-glucose, †*P*<0.03 versus corresponding values in the absence of insulin, apocynin, tempol or Mn(III)TMPyP. Values are mean ± S.E.M. (*n* = 4–7).

D-Glucose increased ROS formation (*SC*_50_ = 9.9 ± 0.5 mmol/L), an effect abolished by apocynin and insulin (Fig 4A). High D-glucose also increased O_2_^•–^ generation, which was abolished by apocynin, insulin and/or tempol (Fig 4B). High D-glucose and insulin increased the NO level in cells in 5 mmol/L D-glucose; however, insulin but not apocynin or tempol blocked the high D-glucose-mediated increase in the NO level (Fig 4C).

### SOD mimetics block D-glucose–reduced BH_4_ level

HUVECs exposed to high D-glucose show lower BH_4_ level compared with cells in 5 mmol/L D-glucose, an effect unaltered by insulin or apocynin (Fig 4D). However, the SOD mimetics tempol and Mn(III)TMPyP increased the BH_4_ level to comparable values in cells in 5 or 25 mmol/L D-glucose.

### Insulin and tempol block D-glucose–increased p42/44^mapk^ phosphorylation

**Fig 5:**
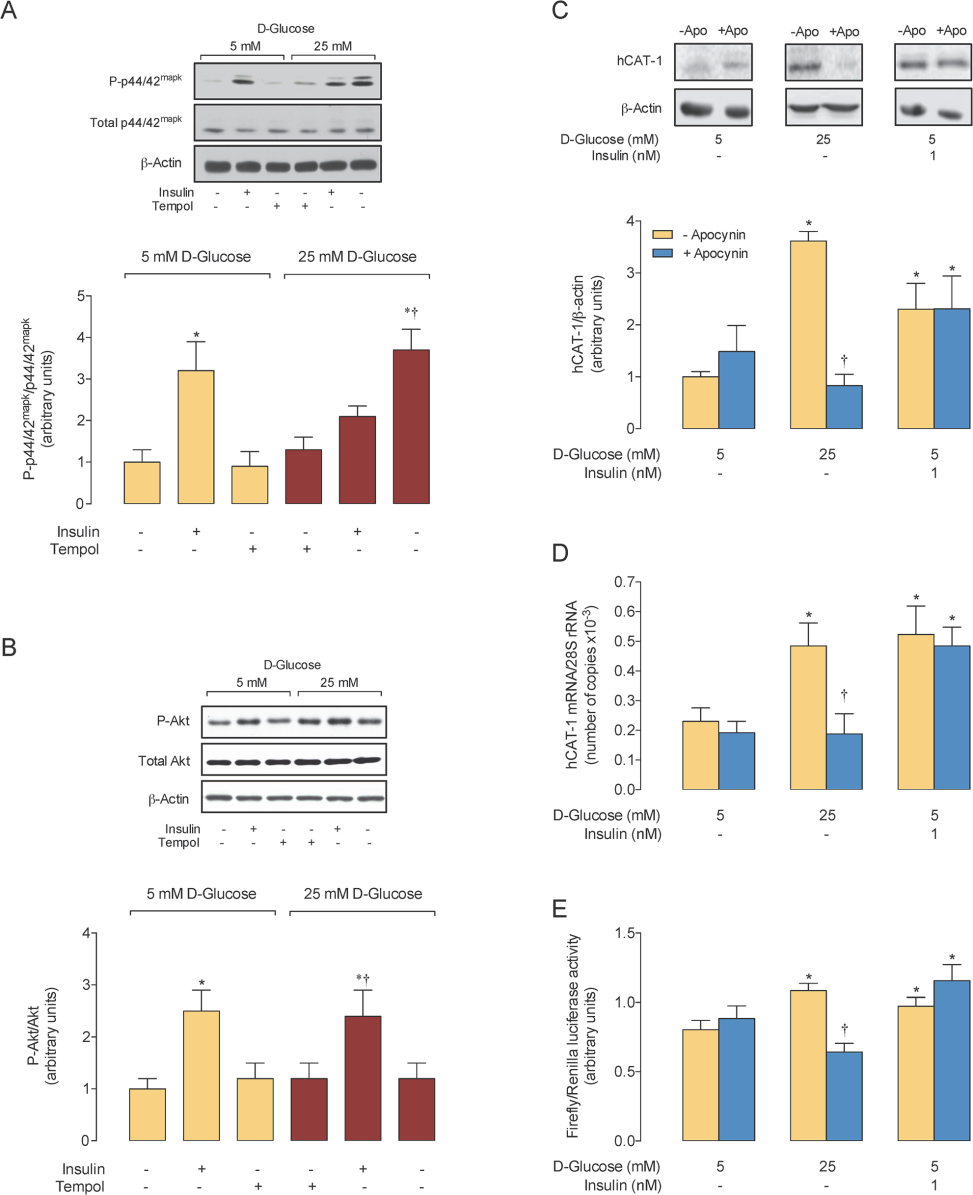
Apocynin blocks high D-glucose, but not insulin-increase in hCAT-1 expression and *SLC7A1* promoter activity. A, Western blot for total (Total p42/44^mapk^) or phosphorylated p42/44^mapk^ (P~p42/44^mapk^) protein abundance in HUVECs incubated (24 hours) in 5 or 25 mmol/L D-glucose in the absence (–) or presence (+) of 1 nmol/L insulin or 1 mmol/L tempol. β -Actin is internal reference. *Lower panel*: P~p42/44^mapk^/Total p42/44^mapk^ ratio densitometries normalized to 1 in 5 mmol/L D-glucose in the absence of insulin or tempol. B, Western blot for total (Total Akt) or phosphorylated Akt (P~Akt) protein abundance as in A. *Lower panel*: P~Akt/Total Akt ratio densitometries normalized to 1 in 5 mmol/L D-glucose in the absence of insulin or tempol. C, Western blot for hCAT-1 protein abundance in the absence (–) or presence of insulin, without (–Apo) (Control) or with (+Apo) 100 μmol/L apocynin. *Lower panel*: hCAT-1/β-actin ratio densitometries normalized to 1 in 5 mmol/L D-glucose in the absence of insulin or apocynin. Bars are cells without (–Apocynin) or with 100 μmol/L apocynin (+Apocynin). D, Number of copies of *hCAT-1* mRNA and *28S* rRNA (internal reference) in cells as in C. E, Luciferase reporter construct activity for cells transfected with a truncated *SLC7A1* promoter (-650 bp (pGL3-hCAT-1^-650^) from ATG) as in C. In A–B, *P<0.04 versus all other corresponding values in 5 or 25 mmol/L D-glucose. †*P*<0.05 versus 5 mmol/L D-glucose in the absence of insulin and tempol. In C–E, *P<0.05 versus corresponding values in the absence of insulin and apocynin. †*P*<0.05 versus corresponding value in 25 mmol/L D-glucose in the absence of apocynin. Values are mean ± S.E.M. (*n* = 5–6).

In cells in 5 mmol/L D-glucose insulin increased p42/44^mapk^ (Fig 5A) and Akt (Fig 5B) phosphorylation, but tempol did not alter the phosphorylation of these molecules under this experimental condition. High D-glucose increased p42/44^mapk^, but not Akt phosphorylation, which was reduced by insulin or tempol.

### Role of NADPH oxidase on hCAT-1 expression

Apocynin blocked the increase in hCAT-1 protein abundance (Fig 5C), *hCAT-1* mRNA expression (Fig 5D), and *SLC7A1* promoter activity caused by high D-glucose in cells transfected with the pGL3-hCAT1^-650^ construct (Fig 5E). However, the increase in these parameters caused by insulin in cells in 5 mmol/L D-glucose was unaltered by this inhibitor.

### Effect of D-glucose on vascular reactivity

**Fig 6:**
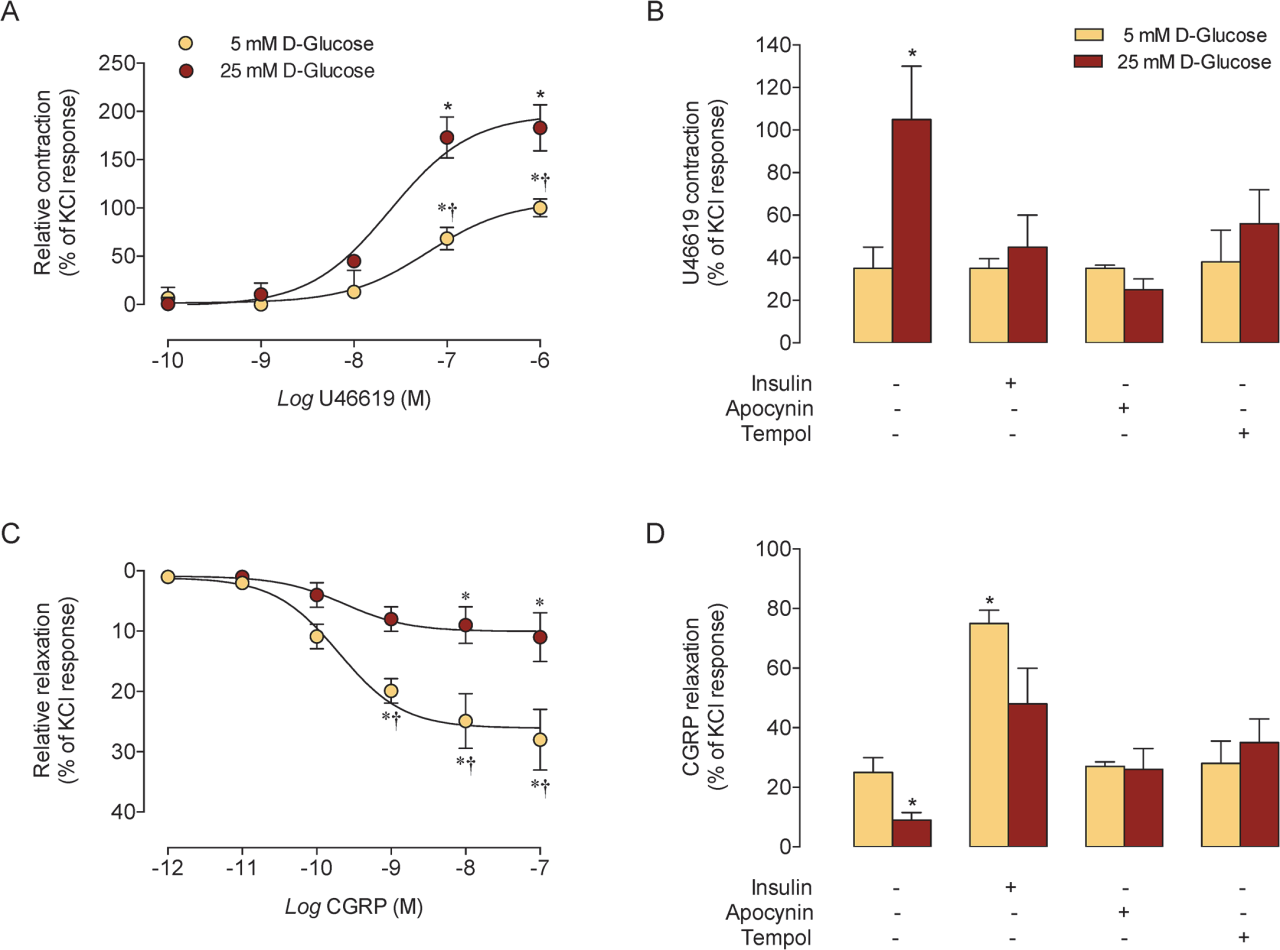
Apocynin, tempol and insulin block high D-glucose effect on human umbilical vein ring reactivity. A, Response of human umbilical vein rings to U46619 incubated (5 minutes) in 5 or 25 mmol/L D-glucose (24 hours). Relative responses are given as a percentage fraction of the initial vessel response to KCl (see Methods). B, Maximal response of umbilical vein rings to 100 nmol/L U46619 as in A in the absence (–) or presence (+) of 1 nmol/L insulin, 100 μmol/L apocynin or 1 mmol/L tempol. C, Response of vein rings to CGRP as in A. D, Maximal response to 10 nmol/L CGRP as in B. In A and C, *P<0.03 versus corresponding values at lower concentrations of either U46619 or CGRP. †*P*<0.05 versus corresponding values in 25 mmol/L D-glucose. In B, *P<0.02 versus all other values. In D, *P<0.02 versus all other corresponding values. Values are mean ± S.E.M. (*n* = 4–7).

Incubation of human umbilical vein rings with high D-glucose for 24 hours caused a higher maximal contraction in response to U46619, compared with vein rings in 5 mmol/L D-glucose (Fig 6A). The *SC*_50_ for the U46619 effect in high D-glucose (24.5 ± 0.6 nmol/L) was lower (*P* < 0.05) than that in 5 mmol/L D-glucose (62.3 ± 0.9 nmol/L). Insulin, apocynin and tempol blocked the U46619-increased contraction in high D-glucose (Fig 6B). Umbilical vein rings in high D-glucose show reduced maximal dilation in response to CGRP compared with vein rings in 5 mmol/L D-glucose (Fig 6C). The half-effective concentration (*EC*_50_) for the CGRP vasodilation was similar in both experimental conditions (9.7 ± 0.2 and 9.6 ± 0.2 nmol/L for 5 and 25 mmol/L D-glucose, respectively). Insulin, but not apocynin or tempol increased vein rings dilation in 5 mmol/L D-glucose; however, these molecules reversed the high D-glucose–decreased vein rings dilation (Fig 6D). In addition, 20 mmol/L D-mannitol + 5 mmol/L D-glucose did not alter vascular reactivity to U46619 or CGRP (not shown).

## Discussion

This study shows that high extracellular D-glucose increases L-arginine transport, NO synthesis and O_2_^•–^ generation through eNOS and NADPH oxidase activation. Additionally, high D-glucose increased the contractile response to U46619 in umbilical vein rings. Insulin reversed these effects of high D-glucose, leading to normal hCAT-1 expression, NO synthesis, O_2_^•–^ generation and vasorelaxation. Insulin and tempol restored high D-glucose–increased p42/44^mapk^ activation. Insulin acts as a protective factor for fetoplacental vascular dysfunction by reducing the oxidative stress caused by high D-glucose.

D-Glucose caused a biphasic increase in L-arginine transport with a half-maximal primary peak stimulation of L-arginine transport (^*pp*^ *SE*_50_) by 10 and 15 mmol/L D-glucose at longer time of incubation (~2-fold) than for 20 and 25 mmol/L D-glucose. Thus, ^*pp*^ *SC*_50_ at higher and lower D-glucose concentrations may result from different mechanism(s). Cells exposed for 24 hours to high D-glucose exhibited a ^*pp*^ *SE*_50_ value similar to that observed upon the increase in hCAT-1 protein abundance, suggesting that high D-glucose-mediated increased L-arginine transport likely occurred due to a higher hCAT-1 level. Because at the half-maximal secondary peak stimulation of transport (^*sp*^ *SC*_50_) values were similar for all D-glucose concentrations, either a mixture of mechanisms or different mechanisms may account for this phenomenon; however, there was still an increase in hCAT-1 protein abundance in HUVECs. Interestingly, increased hCAT-1 expression does not seem to be involved in the increased L-arginine transport reported in response to 25 mmol/L D-glucose for 48 hours in human aortic endothelial cells (HAECs) [25]. Thus, modulation of L-arginine transport by high D-glucose could be different depending on the source of human macrovascular endothelium. High D-glucose also caused a biphasic increase in *hCAT-1* mRNA expression with ^*pp*^ *SE*_50_ and ^*sp*^ *SE*_50_ occurring earlier than for L-arginine transport (72 ± 12 and 78 ± 8 minutes, respectively), and ^*pp*^ *SE*_50_ occurring earlier than hCAT-1 protein abundance (78 ± 7 minutes). No reports are available that address the potential half-life of the hCAT-1 protein and *hCAT-1* mRNA in human endothelial cells [11]. The *CAT-1* mRNA half-life was 90–250 minutes in the rat hepatoma FTO2B cell line [26] and approximately 75 minutes in the stably transfected rat glioma C6 cell line [27]. In addition, *CAT-1* mRNA turnover in response to amino acid deprivation increased between 6–12 hours. Thus, *hCAT-1* mRNA turnover would be approximately 12 hours in response to high D-glucose, with a half-life of 1–2 hours in HUVECs in 5 mmol/L D-glucose in the absence of insulin. After challenge with high D-glucose, the *hCAT-1* mRNA turnover kinetics could be extended to approximately 6–12 hour cycles.

Insulin increases L-arginine transport and NO synthesis involving protein kinase B (PKB)/Akt in HAECs [25]. Incubation of HAECs with 25 mmol/L D-glucose increased L-arginine transport, which is blocked by 1 nmol/L insulin. High D-glucose–increased hCAT-1 protein abundance, and activity in HUVECs was also blocked by insulin; however, the insulin effect was observed at a lower concentration (~10-fold) in HUVECs than in HAECs, with *IC*_50_ close to insulinemia in human umbilical vein (~0.041 nmol/L) [28] and whole umbilical cord (~0.025 nmol/L) [29, 30]. Thus, HUVECs are highly sensitive to insulin compared with HAECs. Insulin also restored the high D-glucose-mediated increase in *V*_max_/*K*_m_, suggesting a lower number of membrane transporters rather than reduced affinity of a fixed number of transporters [11, 18]. Indeed, insulin restored hCAT-1 protein abundance and the plasma membrane/cytosol distribution. Temporality in changes mediated by high D-glucose in hCAT-1 protein abundance and activity are similar; thus, increased transport caused by high D-glucose could result from higher hCAT-1 protein abundance, preferentially localised at the plasma membrane in HUVECs. High D-glucose–associated changes in hCAT-1 expression could result from transcriptional and/or post-transcriptional modulation by insulin. In fact, the insulin effect requires transcriptional regulatory factors acting at the -650 bp fragment of the promoter region of *SLC7A1*. Specific protein 1 (Sp1) mediates insulin effects in several cell types, similar to other TATA-less promoters [11, 31, 32], including increased *SLC7A1* transcriptional activity in HUVECs [12]. *SLC7A1* exhibits at least four Sp1 consensus sequences between -177 and -105 bp from its translation start point in HUVECs [11, 12], making it likely that insulin-restored *SLC7A1* promoter activity results from reduced Sp1 activity. However, other transcription factor(s) that reduce *SLC7A1* transcriptional activity, such as the C/EBP homologous protein 10 (CHOP) as reported in C6 rat glioma cells [33], may be involved in mediating insulin effects.

NADPH oxidase generates ROS in HUVECs exposed to hyperglycemia [18]. We showed that high D-glucose increases ROS generation with a *SC*_50_ value of approximately 11 mmol/L, which is similar to the stimulatory effect by high D-glucose on L-arginine transport (*SC*_50_ of ~13 mmol/L). Thus, the high D-glucose–mediated increase in ROS generation could lead to increased L-arginine transport in HUVECs. Because ROS generation was blocked by apocynin, high D-glucose activation of NAPDH oxidase is feasible, agreeing with findings in human umbilical artery endothelium exposed to 30 mmol/L D-glucose [34] and in EAhy926 cells (immortalised endothelial cell line) exposed to 35 mmol/L D-glucose [35]. Apocynin blocked the high D-glucose-mediated increase in ROS and O_2_^•–^ generation in similar proportions (~69 and ~83%, respectively), suggesting that O_2_^•–^ is the main form of NADPH oxidase-generated ROS (~82%). The latter was further supported by the results showing that the SOD mimetic tempol reduced the effect of high D-glucose in O_2_^•–^ generation. Interestingly, insulin blocked ROS and O_2_^•–^ generation under high D-glucose; thus, a potential anti-oxidative stress role for insulin by decreasing NADPH oxidase activity is feasible in HUVECs, which agrees with findings in mesenteric arterioles from diabetic rats [36]. However, since HUVECs exposed to high D-glucose increases mitochondrial O_2_^•–^ generation [37], the possibility that insulin also modulates this source of O_2_^•–^ generation is likely.

Insulin and tempol reversed the high D-glucose–increase in p42/44^mapk^ phosphorylation, suggesting that cell signalling mediated by activation of these protein kinases could result from a higher O_2_^•–^ generation under high D-glucose. In addition, a potential protective effect of insulin in this phenomenon is feasible. On the contrary, since high D-glucose did not alter Akt phosphorylation it is likely that this molecule is not involved in the response to this environmental condition enriched in O_2_^•–^ in HUVECs from normal pregnancies. The insulin response in this study is similar to that reported in primary cultures of HUVECs from GDM pregnancies [38]. Since GDM associates with overexpression of insulin receptor A (IR-A) form in HUVECs, and activation of these receptors leads to preferential p42/44^mapk^ phosphorylation compared with Akt phosphorylation in HUVECs from normal pregnancies, it is feasible that insulin mediates similar mechanisms to restore p42/44^mapk^ associated cell signalling in a high D-glucose environment and in GDM.

High D-glucose caused comparable increases in NO and ROS generation; thus, these phenomena are linked in HUVECs, agreeing with a report on this cell type when it was incubated with 75 mmol/L D-glucose [39]. The high D-glucose-mediated increase in NO synthesis is NADPH oxidase-independent, as apocynin was ineffective. However, because insulin blocked the D-glucose effect on NO synthesis and ROS generation, an alternative mechanism for the response to this hormone in HUVECs is likely. Thus, insulin could be equally active in preventing O_2_^•–^ oxidative stress in HUVECs under high D-glucose conditions. eNOS uncoupling due to imbalanced tetrahydrobiopterin (BH_4_) and dihydrobiopterin (BH_2_) levels (BH_2_/BH_4_ > 1) leads to O_2_^•–^ generation [40]. Paradoxically, even when high D-glucose causes an increase in the NO level, our results also show that BH_4_ level is lower under this condition, supporting the possibility that at least a fraction of eNOS could be uncoupled in high D-glucose. Since only the SOD mimetics tempol and Mn(III)TMPyP increased the BH_4_ level to comparable values in cells in 5 or 25 mmol/L D-glucose it is likely that O_2_^•–^ generation by HUVECs in both of these experimental conditions maintains a lower expression of this cofactor. In fact, O_2_^•–^ generated by NADPH oxidase is unlikely since apocynin did not reverse the reduced BH_4_ level detected in cells in high D-glucose. Thus, an alternative to NADPH oxidase-generated O_2_^•–^ by high D-glucose is eNOS uncoupling in HUVECs. The latter hypothesis is further supported by (a) a better correlation between the NO level and O_2_^•–^ generation observed in response to 25 mmol/L compared with 5 mmol/L D-glucose ((NO/O_2_^•–^)^25 mmol/L^/(NO/O_2_^•–^)^5 mmol/L^ = 1.22), (b) increased NADPH-dependent O_2_^•–^ generation by reducing the eNOS dimer/monomer ratio (i.e., uncoupled eNOS) and NO synthesis in EA.hy926 cells exposed to 35 mmol/L D-glucose [35], (c) restoration of eNOS dimerization and function by BH_4_ administration in diabetic rats [36] and (d) restoration by apocynin of the diabetes mellitus-increased eNOS-derived O_2_^•–^ in mice [41]. Taking in consideration that incubation of HUVECs with ~22.5 mmol/L D-glucose causes an estimated increase of ~2.3 fold of the peroxynitrite (ONOO^–^) level [42], a metabolite that results from the reaction between NO and O_2_^•–^, and since in our study the increase in NO level (~1.8 fold) and O_2_^•–^ generation (~2.1 fold) were similar, we speculate on the possibility that ONOO^–^ generation is likely increased in ~2 fold in HUVECs exposed to high D-glucose. In addition, insulin caused a comparable reduction (~32%) in NO level and O_2_^•–^ generation in cells in high D-glucose, suggesting the possibility that this hormone could also cause a reduction in ONOO^–^ generation in HUVECs. However, since apocynin or tempol restored the high D-glucose–elevated O_2_^•–^ generation, but not the increase in the NO level to values in cells in 5 mmol/L D-glucose, O_2_^•–^ generation from NADPH oxidase may not be enough to cause an increase in the ONOO^–^ generation in high D-glucose in this cell type.

Increases in hCAT-1 protein abundance, *hCAT-1* mRNA expression and *SLC7A1* promoter activity by high D-glucose, but not insulin, were blocked by apocynin, suggesting a NADPH oxidase-dependent mechanism. It has been reported that eNOS activity is associated with higher hCAT-1–mediated L-arginine transport in EA.hy926 cells [13]. Because insulin blocked the increase in *V*_max_/*K*_m_ for L-arginine transport but reduced the high-D-glucose-mediated increase in the NO level by ~63%, the high D-glucose effect was partially dependent (~25–30%) on L-arginine transport in HUVECs. In addition, lower hCAT-1 protein abundance compared with reduced *V*_max_/*K*_m_ could be mainly responsible (~75%) for the insulin-mediated reversal of NO synthesis. Our findings also show that insulin reduced hCAT-1 protein abundance at the plasma membrane at high D-glucose concentrations. Because insulin increased hCAT-1 protein abundance in membrane fractions in HUVECs in 5 mmol/L D-glucose [12] and caused a general increase in its cellular distribution in this cell type, reduced hCAT-1 protein abundance at the plasma membrane in cells challenged with high D-glucose could be caused by insulin. These findings support the possibility that not only a reduction in the total protein abundance but also potential hCAT-1 redistribution results from incubation with high D-glucose and insulin. The opposite response to insulin regarding hCAT-1 activity and expression in HUVECs in 5 mmol/L versus 25 mmol/L D-glucose is a finding that is similar to the differential response to this hormone reported for eNOS expression and activity as well as for the human equilibrative nucleoside transporter 1 (hENT1) expression and activity in HUVECs from normal pregnancies compared with GDM pregnancies [38]. The mechanisms behind these opposite effects of insulin involve activation of A_1_ and A_2A_ adenosine receptors leading to modulation of the biological effect of insulin in HUVECs from normal pregnancies [18], GDM pregnancies [38], and in preeclampsia [43]. These mechanisms were not evaluated in this study; however, it is suggested that insulin could use an intracellular metabolic machinery to modulate expression of *SLC29A7* and hCAT-1 protein that is different in HUVECs in a normal or high D-glucose environment, as reported in GDM [44].

Umbilical vein contraction was caused by the thromboxane A_2_ mimetic U46619, as reported in human chorionic plate arteries [24]. The U46619 maximal contraction in high D-glucose conditions was higher (~1.8-fold) compared with 5 mmol/L D-glucose, suggesting that reduced bioavailability of agents causing vasorelaxation or increased agents causing vasoconstriction may be responsible for this phenomenon. Interestingly, U46619 half-maximal constriction in high D-glucose was higher than in 5 mmol/L D-glucose (^25 mmol/L^ *SC*_50_/^5 mmol/L^ *SC*_50_ = 2.5), suggesting that under high D-glucose, umbilical vein rings are more reactive to this molecule than preparations in a physiological concentration of D-glucose. It is likely that increased contraction of umbilical vein rings in high D-glucose conditions occurred due to increased ROS and O_2_^•–^ generation because this vascular response was blocked by insulin, apocynin and tempol. Since similar results were found in the response of umbilical vein rings to CGRP (a preferential endothelium-dependent vasodilator), not only constriction, but also dilation are altered involving similar mechanisms in terms of oxidative stress in vein rings exposed to high D-glucose. Interestingly, since CGRP half-maximal dilation in high D-glucose was similar to that in 5 mmol/L D-glucose (^25 mmol/L^ *EC*_50_/^5 mmol/L^ *EC*_50_ = 1.02), a reduced sensibility to vasodilators is unlikely to be the cause of the high D-glucose–increased vasoconstriction in human umbilical vein rings.

**Fig 7:**
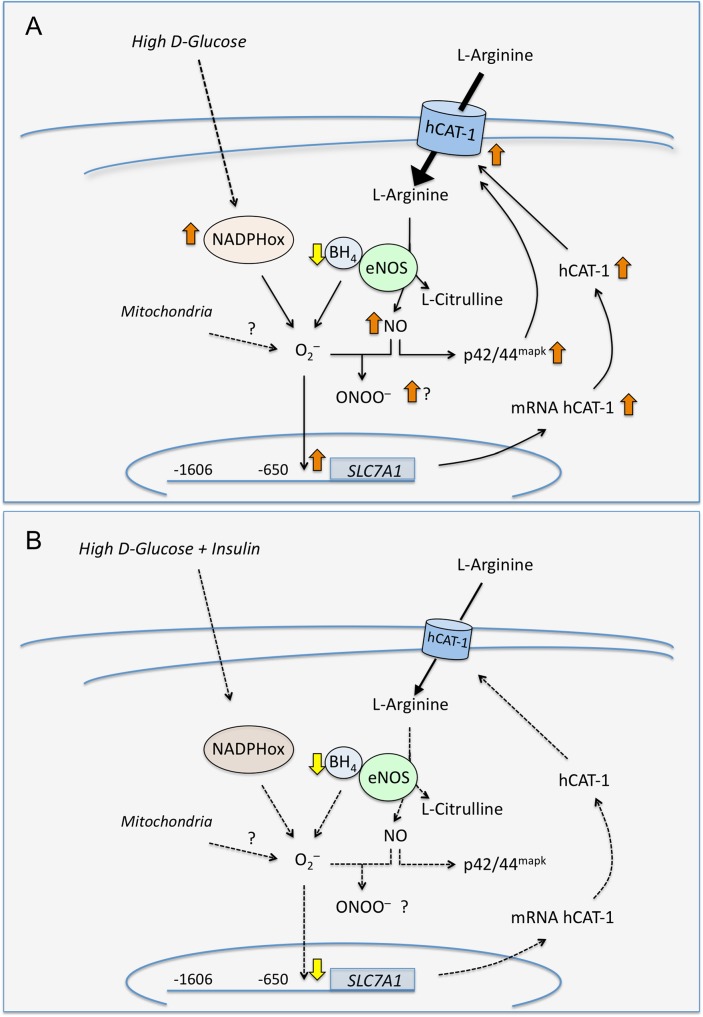
Involvement of high D-glucose–increased oxidative stress on L-arginine transport in human umbilical vein endothelium. A, Exposure of HUVECs to 25 mmol/L D-glucose (*High D-glucose*) leads to an increase (⇧) in the plasma membrane abundance of the human cationic amino acid transporter 1 (hCAT-1) protein resulting in a higher L-arginine uptake (solid black arrow). High D-glucose activates (⇧) NADPH oxidase (NADPHox) leading to higher generation of reactive oxygen species, including superoxide anion (O_2_^•–^). In addition, high D-glucose increases nitric oxide (NO synthesis from endothelial NO synthase (eNOS) leading to formation of L-citrulline. High D-glucose also increases O_2_^•–^ generation likely via uncoupled eNOS due to reduced (⇩) tetrahydrobiopterin level (BH_4_). Increased O_2_^•–^ generation most likely (?) leads to formation of peroxynitrite (ONOO^–^) from nitric oxide (NO) reducing the NO bioavailability. NO also activates p42/44^mapk^. These phenomena result in increased *SLC7A1* promoter activity up to -650 bp from the ATG due to higher O_2_^•–^ generation, with higher hCAT-1 mRNA and protein abundance. B, Insulin reduces (dashed arrows) O_2_^•–^generation to values in cells in 5 mmol/L D-glucose (i.e., a normal D-glucose concentration) leading to normal hCAT-1 expression (*SLC7A1* promoter expression, hCAT-1 mRNA and protein abundance) and transport activity, and NO synthesis. However, insulin did not restore the reduced BH_4_ level detected in HUVECs in high D-glucose. The changes caused by high D-glucose result in higher vasoconstriction and reduced vasodilation associated with increased oxidative stress due to NADPH oxidase activation and/or eNOS uncoupling in HUVECs.

HUVECs exposure to high D-glucose increases L-arginine transport (Fig 7), likely resulting from higher hCAT-1 expression and protein abundance in the plasma membrane. This mechanism could be an adaptive response of HUVECs to higher ROS and O_2_^•–^ generation from high D-glucose–activated NADPH oxidase. In parallel, high D-glucose increased NO synthesis, which was independent of NADPH oxidase activation. Insulin reversed the high D-glucose-mediated alterations in L-arginine transport involving the modulation of *SLC7A1* gene expression, leading to altered umbilical vein reactivity. Modulation of hCAT-1 expression and activity is key to maintaining umbilical vein tone and endothelial function. Most diseases of pregnancy progress with oxidative stress leading to altered placental vascular reactivity [11]. Thus, management of potential pro-oxidative stress conditions is necessary to prevent fetoplacental endothelial dysfunction, thus ensuring an adequate supply of nutrients to the growing foetus.
